# Anticancer Effects of Combined Blue Light and Ionizing Irradiation

**DOI:** 10.3390/cimb48010045

**Published:** 2025-12-29

**Authors:** Keita Kitano, Hironori Yoshino, Kosuke Kawanami, Ryosuke Kajimoto, Eichi Tsuruga

**Affiliations:** 1 Department of Radiation Science, Hirosaki University Graduate School of Health Sciences, Hirosaki 036-8564, Aomori, Japan; h25mh204@hirosaki-u.ac.jp (K.K.); tsuru@hirosaki-u.ac.jp (E.T.); 2 Department of Radiological Technology, Hirosaki University School of Health Sciences, Hirosaki 036-8564, Aomori, Japan

**Keywords:** blue light, anticancer effect, head and neck squamous cell carcinoma cells

## Abstract

While there have been reports indicating the potential anticancer benefits of blue light irradiation and its enhanced effectiveness when combined with anticancer drugs, no studies have explored its combined use with radiation therapy. In this study, the anticancer effects of blue light irradiation alone and in combination with radiation therapy were investigated in vitro. Blue light was applied using a transilluminator (470 nm). For combination experiments, cells were exposed to X-rays 24 h after blue light irradiation. Cell viability was assessed using the trypan blue exclusion method, and protein expression was analyzed by Western blotting. Blue light irradiation suppressed the proliferation of human head and neck squamous cell carcinoma (HNSCC) cells. Furthermore, combined blue light and X-ray irradiation more effectively inhibited the proliferation of human HNSCC cells compared to either irradiation alone. Mechanistically, the irradiation of HNSCC cell line SAS with blue light suppressed the activity of extracellular signal-regulated kinase (ERK1/2), which is an important kinase that is involved in cell proliferation. Collectively, these findings suggest that blue light suppresses the proliferation of HNSCC cells, at least in part through ERK1/2 inactivation observed in SAS cells, and that its combination with radiation may represent a promising therapeutic approach.

## 1. Introduction

Radiation therapy is one of the major treatments for cancer and is used for various cancer types, such as lung cancer and cancers of the head and neck. Although radiation therapy is highly effective for localized treatment, it is impossible to avoid irradiating healthy organs adjacent to the tumor tissue, potentially leading to serious tissue damage. To prevent or reduce such tissue damage associated with radiation therapy, a strategy is needed to reduce the prescribed radiation dose while maintaining the lethal effect on the tumor by combining radiation with other local therapies.

Visible light changes color depending on its wavelength. For instance, red light has a wavelength of 660 nm, while blue light has a wavelength of 470 nm. Furthermore, it is generally considered that shorter wavelengths correspond to higher energy. Recently, visible light applications in medicine have increased [1]. For example, red light is utilized to relieve pain associated with orthodontic treatment since it reduces pain and inflammation while promoting the healing process of tissues [2]. As for blue light, it is utilized for acne treatment due to its bactericidal effect [3]. It is also used for wound healing in the skin [4].

In addition, recent studies have explored the use of blue light in the treatment of cancer. For example, Young et al. demonstrated in vitro that blue light irradiation inhibits the proliferation of pancreatic cancer cells [5]. As reported by Yang et al., blue light irradiation on human osteosarcoma MG63 cells also exhibits anticancer effects through apoptosis induction [6]. Nevertheless, research on the anticancer effects of blue light irradiation remains scarce, and further verification is needed.

In addition, the combined effects of blue light and existing anticancer drugs have been explored. When blue light irradiation was used alongside two types of anticancer agents—the anti-apoptotic protein inhibitor AT406 [7,8,9] and the nuclear factor kappa-B inhibitor Rocaglamide [10]—against human colon cancer HCT116 and HT29 cells, it was expected that this combination would yield more potent anticancer effects compared to using either treatment alone [11], potentially allowing for reduced drug dosages and mitigating side effects. Therefore, the combination of blue light irradiation and existing cancer treatments is expected to be promising. However, there are limited studies regarding the use of blue light irradiation in combination with existing cancer therapies, and, specifically, there are no reports on its combination with radiation therapy.

The anticancer effects of blue light irradiation alone and in combination with ionizing radiation on human cancer cell lines in vitro are evaluated in this study, and the underlying mechanisms are investigated as well.

## 2. Materials and Methods

### 2.1. Reagents

PBS(−) (Ca^2+^, Mg2^+^-free Dulbecco’s phosphate-buffered saline), trypsin, methanol, trypan blue, and Giemsa staining solution were procured from Wako Pure Chemical Industries (Osaka, Japan). Propidium iodide (PI) and dimethyl sulfoxide (DMSO) were purchased from Sigma-Aldrich (St. Louis, MO, USA). Anti-GAPDH rabbit antibody (#5174), anti-p42/44 rabbit-derived antibody (#4695), anti-phospho-p42/44 rabbit antibody (#4370), and anti-cleaved caspase3 rabbit antibody (#9661) were supplied by Cell Signaling Technology Japan, K.K. (Tokyo, Japan). SCH772984 (19166) was purchased from Funakoshi (Tokyo, Japan). CM-H_2_DCFDA (DCF; C6827) was purchased from Molecular Probes (Thermo Fisher, Scientific, Inc., Eugene, OR, USA). FITC-conjugated Annexin V (FITC-Annexin V) and Annexin V binding buffer were purchased from BioLegend (San Diego, CA, USA).

### 2.2. Cell Culture and Treatment

Human head and neck squamous cell carcinoma (HNSCC) cell lines SAS and Ca9-22, human colon cancer cell line HCT116, and human lung cancer cell A549 were obtained from RIKEN Bio-Resource Center (Tsukuba, Japan). SAS and Ca9-22 were cultured in Dulbecco’s Modified Eagle Medium (DMEM) high-glucose (Wako) medium containing 10% fetal bovine serum (FBS) and 1% penicillin–streptomycin (P/S) (Wako). HCT116 and A549 were maintained in DMEM low-glucose (Wako) medium that contains 10% FBS and 1% P/S. All cells were cultured at 37 °C in a humidified atmosphere containing 5% CO_2_.

HUVECs (Catalog No. 200-05n) were purchased from Cell Applications, Inc. (San Diego, CA, USA). HUVECs were seeded onto collagen I-coated dishes (35 mm; Iwaki, Tokyo, Japan) and cultured with Endothelial Cell Growth Medium Kit (C-22110, Takara Bio, Inc., Kusatsu, Shiga, Japan).

Cells were seeded at 5 × 10^4^ cells/well in a 12-well plate (Sumitomo Bakelite Co., Ltd., Tokyo, Japan). The following day after seeding, cells were irradiated with blue light, and after irradiation, the cells were collected and used for each analysis. In the combined radiation experiment, X-ray irradiation was performed 24 h after blue light irradiation. In order to ensure that the time spent outside the CO_2_ incubator was consistent across all experimental conditions, the cells were incubated at room temperature.

Cells were seeded at 5 × 10^4^ cells/well in a 12-well plate, and extracellular signal-regulated kinase (ERK1/2) inhibitor SCH772984 (1 or 5 μM) was added the following day. After 2 days, cells were harvested, and viable cell numbers were quantified using the trypan blue dye exclusion assay.

### 2.3. Blue Light Irradiation

**Figure 1:**
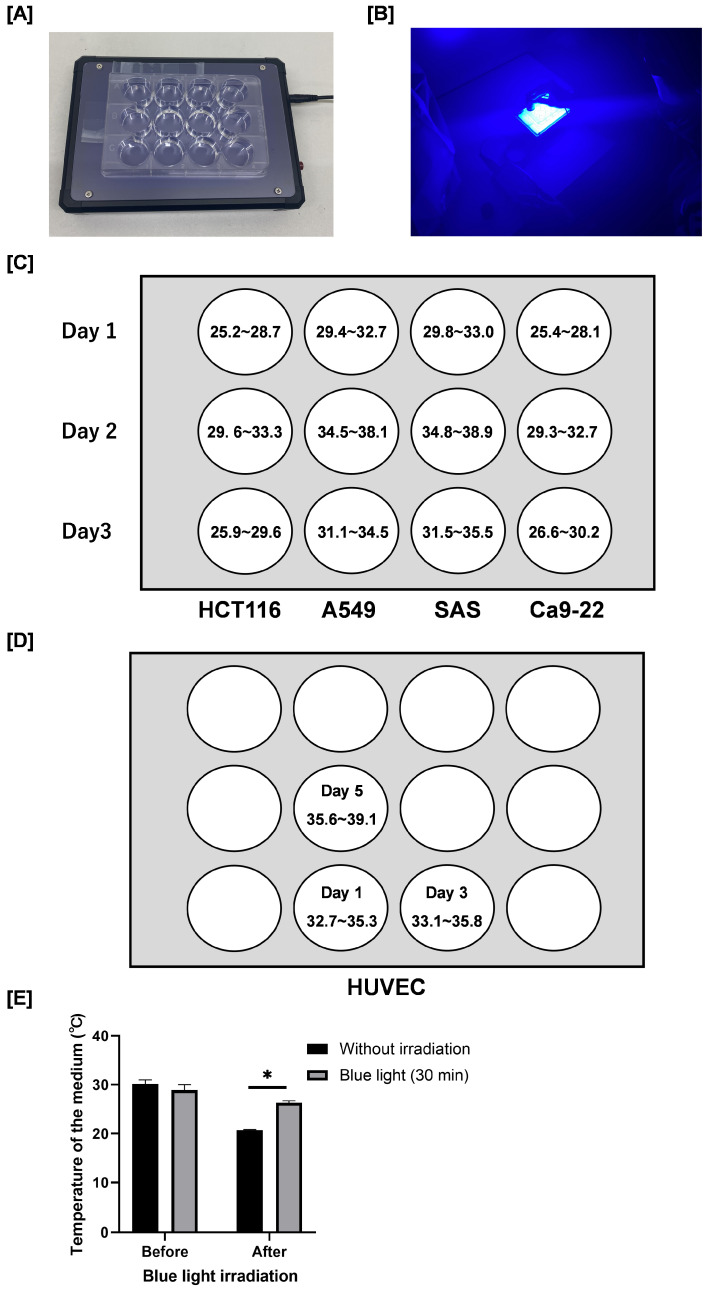
Blue light transilluminator irradiation method and the irradiance. (**A**) Blue light irradiation was carried out by using a blue light transilluminator (LEDB-SBOXHP) manufactured by Optocode Co. (**B**) Blue light irradiation. Each cancer cell was seeded into a 12-well plate as shown in the figure. (**C**,**D**) The radiation intensity [W/m^2^] in each well is also shown. (**E**) Plates containing culture medium (1.0 mL) without cells were incubated at 37 °C for 24 h. After removal from the incubator, the temperature of the medium was immediately measured using a digital thermometer. Following blue light irradiation for 30 min at room temperature, the temperature of the medium was immediately measured again. Data are presented as the mean ± standard error of three independent experiments. * *p* < 0.05.

Blue light irradiation was carried out using a blue LED transilluminator (LEDB-SBOXHP, Optocode Co., Tokyo, Japan, Figure 1A,B) that emits blue light at 470 nm. The irradiance of the blue LED light was measured using an irradiance meter (model number: HD2302.0, Delta Ohm Co., Padova, Italy) and an irradiance light probe (model number: LP471RAD, Delta Ohm Co., Padova, Italy). Figure 1C,D shows the irradiance in each well. The device has a fixed output intensity according to its specifications, and the irradiance cannot be adjusted. Because only a single device was available in our laboratory, it was technically difficult to examine multiple intensity or duration conditions in parallel.

We measured the temperature of the culture medium in the absence of cells using a digital thermometer (MWT-1; MonotaRO Co., Ltd., Osaka, Japan). After removal from the incubator, the temperature of the medium decreased from approximately 30 °C to 21 °C (Figure 1E). Importantly, although the temperature of the blue light–irradiated group (30 min) was significantly higher than that of the non-irradiated control group (26 °C vs. 21 °C), it remained lower than the temperature at the time the culture plates were removed from the incubator. Therefore, we consider that the temperature changes observed under our experimental conditions are unlikely to have significantly influenced the results.

### 2.4. In Vitro X-Ray Irradiation

Cells were irradiated with X-rays using an X-ray generator (MBR-1520R-3; Hitachi, Ltd., Tokyo, Japan) at a source-to-sample distance of 450 mm and at a dose rate of 0.99–1.03 Gy/min (150 kVp; 20 mA; 0.5 mm Al filter and 0.3 mm Cu filter).

### 2.5. Colony Formation Assay

Cells were harvested 24 h after X-ray irradiation, and the number of viable cells was determined. An appropriate number of cells for each condition was seeded in a 60 mm diameter dish (Sumitomo Bakelite Co., Ltd., Tokyo, Japan) and cultured for 8–12 days. After culturing, dishes were washed with PBS(−), fixed with methanol, and stained with Giemsa solution. Colonies containing more than 50 cells were counted. All experiments were performed in triplicate. The surviving fraction was calculated as previously described [12].

### 2.6. SDS-PAGE and Western Blotting

SDS-PAGE and Western blot analysis were carried out as previously reported [13]. The following primary antibodies diluted in Can Get Signal^®^ Immunoreaction Enhancer Solution 1 (Toyobo, Co., Ltd., Osaka, Japan) were used: anti-phospho-p42/44 antibody (1:3000), anti-p42/44 antibody (1:3000), anti-cleaved caspase-3 antibody (1:3000), and anti-GAPDH antibody (1:4000). After being incubated overnight at 4 °C, the membrane was incubated with a secondary antibody (1:10,000) that was diluted in Can Get Signal^®^ Immunoreaction Enhancer Solution 2 (Toyobo, Co., Ltd., Osaka, Japan) for 1 h at room temperature and detected using chemiluminescence with Clarity Western ECL Substrate (Bio-Rad Laboratories, Inc., Hercules, CA, USA). Images were captured using the iBright 1500 Image system (Thermo Fisher Scientific, Inc., Eugene, OR, USA).

### 2.7. Analysis of Intracellular Reactive Oxygen Species

By using flow cytometry with the reactive oxygen species (ROS) detection fluorescent probe DCF, intracellular ROS were analyzed. After treatment, cells were treated with 10 µM DCF for 30 min in a CO_2_ incubator at 37 °C. After this, the cells were rinsed with PBS(−) and harvested. After centrifuging the cells in PBS(−) (1200 rpm, 5 min, 4 °C), the fluorescence intensity of DCF was analyzed using a flow cytometer (CytoFLEX, Beckman-Coulter, Inc., Brea, CA, USA).

### 2.8. Analysis of Apoptosis

Apoptosis was analyzed using FITC-Annexin V and PI staining according to the manufacturer’s instructions. Briefly, cells were harvested, washed twice with PBS(−), centrifuged at 1200 rpm for 5 min at room temperature, and resuspended in 100 μL of Annexin V binding buffer. Subsequently, 5 µL each of FITC-annexin V (90 μg/mL) and PI (1 mg/mL) were added, and the cells were incubated for 15 min at room temperature in the dark. Following incubation, Annexin V binding buffer was added, and samples were analyzed by a flow cytometer.

### 2.9. Statistical Analysis

All quantitative data are presented as the mean ± standard error of three independent experiments. Prior to statistical analysis, data distribution was assessed for normality using the chi-square goodness-of-fit test, and no significant deviation from normality was observed. One sample t-test was carried out using GraphPad prism, and the control group was considered as 100%. Using two-tailed Student’s t-test, a comparison of the two groups was performed. Comparisons of data from multiple groups were subjected to one-way analysis of variance followed by Tukey–Kramer test. A *p*-value of <0.05 was considered significant. Statistical analysis was performed using Excel (Microsoft 365, Washington, DC, USA) with the add-in software Statcel4 (The Publishing OMS Ltd., Tokyo, Japan).

## 3. Results

### 3.1. Effects of Blue Light Irradiation on Human Cancer Cell Proliferation

**Figure 2:**
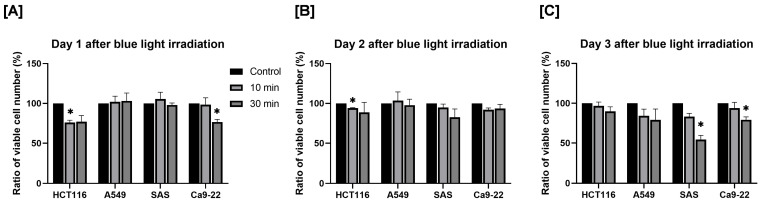
Effect of blue light irradiation on human cancer cell proliferation. Each cell was irradiated with blue light for 10 or 30 min, and the number of viable cells was determined by using the trypan blue exclusion method 1 day (**A**), 2 days (**B**), and 3 days (**C**) after irradiation. The results are shown as relative values to the number of viable cells in non-irradiated cells. * *p* < 0.05 vs. control.

First, we evaluated the effect of blue light irradiation on the proliferation of human cancer cells. Cell proliferation was assessed by determining the relative survival rate, with the number of viable cells in the non-irradiated control group defined as 100%. After 1 day, a 10 min blue light exposure significantly reduced the number of HCT116 cells, and a 30 min exposure significantly reduced the number of Ca9-22 cells (Figure 2A). After 2 days, the number of viable HCT116 cells was significantly decreased following 10 min of irradiation (Figure 2B). After 3 days, the number of SAS and Ca9-22 cells decreased significantly to approximately 50% and 75% of control levels, respectively, after 30 min of irradiation (Figure 2C).

### 3.2. Effects of Combined Exposure to Blue Light and Radiation on Human Cancer Cell Proliferation

Next, in order to investigate the combined effects of blue light and radiation on cancer cell proliferation, cells were first exposed to blue light and then cultured for 24 h, which is followed by X-ray irradiation. After an additional 2 days of culture, viable cell numbers were quantified, and relative survival rates were calculated using the non-irradiated control group as a reference.

**Figure 3:**
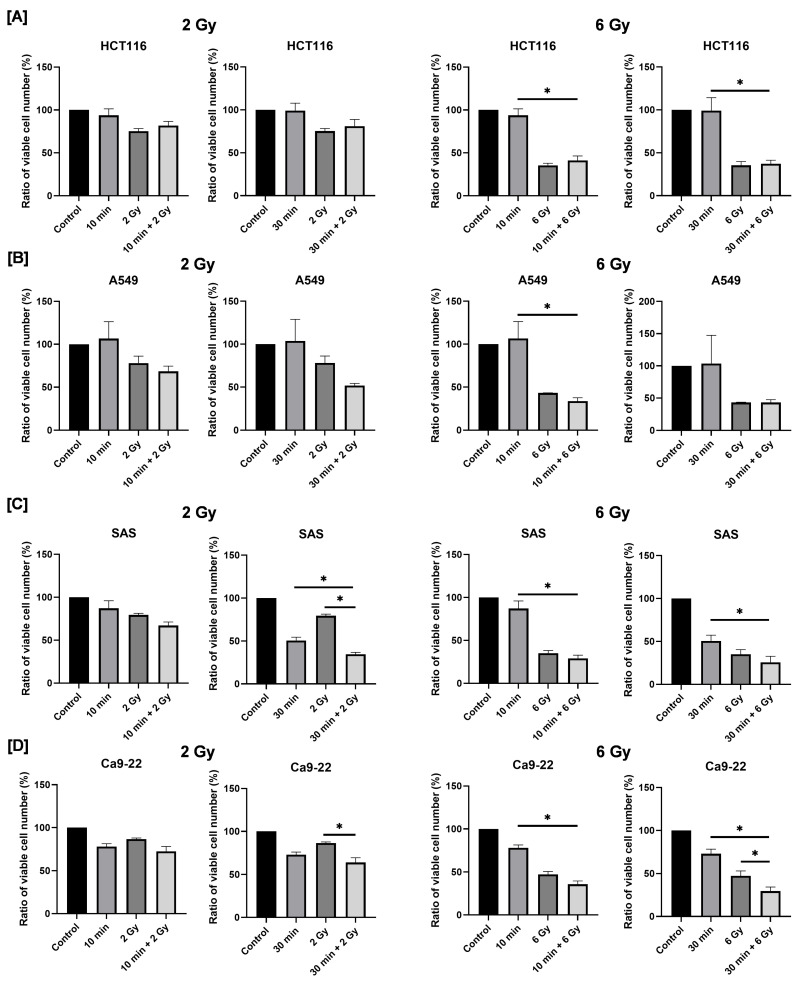
Effects of combined exposure to blue light and X-rays on the proliferation of human cancer cells. HCT116 (**A**), A549 (**B**), SAS (**C**), and Ca9-22 cells (**D**) were irradiated with blue light, and X-rays were irradiated 24 h following blue light irradiation. The number of viable cells was counted 2 days after X-ray irradiation. The results are shown as relative values to the number of viable cells of the non-irradiated cells with blue light and X-rays. * *p* < 0.05.

In HCT116 and A549 cells, no significant difference was observed in cell numbers between the X-ray-only group and the combined irradiation group (Figure 3A,B). In contrast, as shown in Figure 3C, combined exposure to blue light and 2 Gy X-rays resulted in a significantly lower number of viable cells compared with either treatment alone (Figure 3C). Notably, the degree of growth inhibition observed in the combined treatment group was comparable to that induced by 6 Gy irradiation alone. In Ca9-22 cells, combined treatment with blue light and 6 Gy significantly reduced the number of viable cells on day 2 post-irradiation compared with each single-treatment group (Figure 3D). These findings indicate that combined irradiation with blue light and X-ray significantly suppresses the growth of human HNSCC cells, although this effect is cell line-dependent.

### 3.3. Effects of Combined Exposure to Blue Light and Radiation on Intracellular ROS Levels in HNSCC Cells

**Figure 4:**
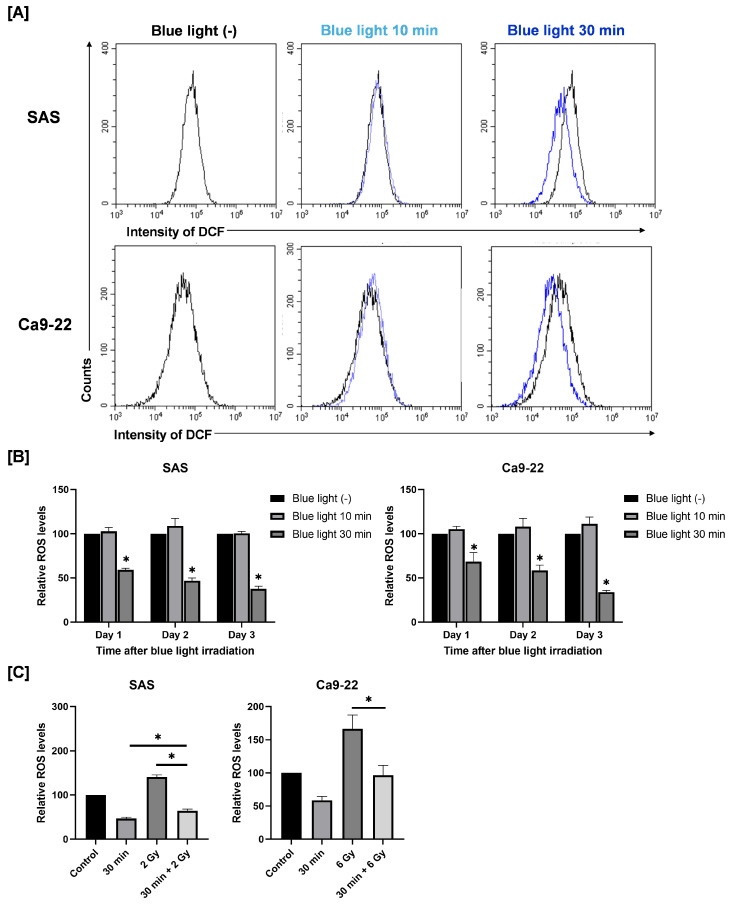
Effects of blue light and/or X-ray irradiation on intracellular ROS levels in SAS and Ca9-22 cells. (**A**,**B**) SAS and Ca9-22 cells exposed to blue light were cultured for 1–3 days and harvested for the analysis of ROS levels. (**A**) A representative histogram of DCF is shown. (**B**) Relative ROS values to cells not exposed to blue light are shown. * *p* < 0.05 vs. Blue LED (−). (**C**) SAS and Ca9-22 cells were exposed to blue light, and then followed by X-ray irradiation 24 h later. Cells were harvested for the analysis of ROS levels at 24 h after X-ray irradiation. The relative ROS values to cells not exposed to blue light are shown. * *p* < 0.05.

Because intracellular ROS play a key role in cellular responses such as cell death [14], we next examined whether blue light affects ROS levels in SAS and Ca9-22 cells. As shown in Figure 4A,B, blue light irradiation for 30 min reduced intracellular ROS levels at 24 h after blue light irradiation. This reduction persisted for up to 2–3 days (Figure 4B). On the contrary, 2 Gy irradiation increased ROS levels (Figure 4C). Importantly, pre-irradiation with blue light significantly suppressed the radiation-induced increase in ROS levels (Figure 4C).

### 3.4. Involvement of ERK1/2 on the Anticancer Effects of Blue Light and in Combination with Ionizing Radiation in HNSCC Cells

Given that ROS is an upstream regulator of ERK1/2 signaling [15], we next examined the involvement of ERK1/2 in the anticancer effects of blue light and its combination with ionizing radiation. ERK1/2 plays a key role in the regulation of numerous processes, such as cell adhesion, cell cycle progression, cell migration, cell survival, differentiation, metabolism, proliferation, and transcription. As ERK1/2 is abnormally activated in cancer cells, inhibiting its activity is considered a crucial strategy for combating tumors [16].

**Figure 5:**
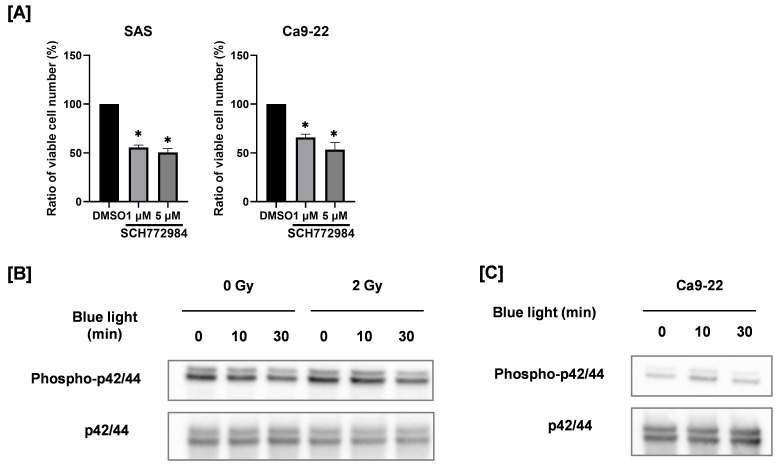
Involvement of ERK1/2 on the anticancer effects by blue light and in combination with ionizing radiation. (**A**) SAS and Ca9-22 cells were cultured for 2 days in the presence of the ERK1/2 inhibitor SCH772984, and the number of viable cells was counted. The results are shown as relative values to the solvent control group (DMSO). * *p* < 0.05 vs. DMSO. (**B**) SAS cells exposed to blue light were irradiated with 2 Gy after 24 h of blue light irradiation. The cells were harvested for Western blotting, 2 days after X-ray irradiation. Representative immunoblot of phosphorylated ERK1/2 are shown. ERK1/2 was used as a loading control. (**C**) Ca9-22 cells exposed to blue light were cultured for 3 days, and the protein expression of phosphorylated ERK1/2 and ERK1/2 was analyzed by Western blotting.

As shown in Figure 5A, the ERK1/2 inhibitor SCH772984 decreased the number of viable HNSCC cells, thus confirming the role of ERK1/2 on the proliferation of HNSCC cells. Therefore, we analyzed the expression of phosphorylated ERK1/2, the active form of ERK1/2. Exposure to blue light for 30 min reduced the expression level of phosphorylated ERK1/2 both in the absence and the presence of 2 Gy irradiation, as shown in Figure 5B. In contrast, blue light irradiation did not affect ERK1/2 phosphorylation in Ca9-22 cells (Figure 5C). These findings indicate that suppression of ERK1/2 activation may contribute to the antiproliferative effects of blue light in a cell line–specific manner.

### 3.5. Apoptosis-Inducing Effects of Blue Light Alone or in Combination with Ionizing Radiation in HNSCC Cells

To determine whether apoptosis contributes to the observed antiproliferative effects, we evaluated apoptotic cell death in HNSCC cells.

**Figure 6:**
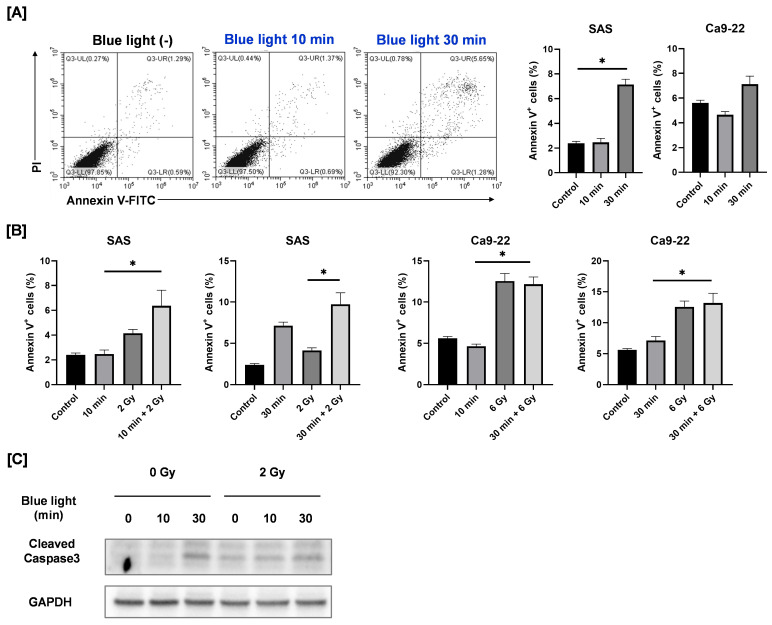
Apoptosis-inducing effects of blue light and in combination with ionizing radiation in HNSCC cells. (**A**) HNSCC cells were irradiated with blue light, cultured for 3 days, and then harvested for the apoptosis analysis. The figure shows representative flow cytometry plots of Annexin V/PI staining of SAS cells under blue light irradiation alone, along with the percentage of Annexin V–positive cells (the sum of Annexin V^+^/PI^−^ cells and Annexin V^+^/PI^+^ cells). * *p* < 0.05. (**B**) HNSCC cells were exposed to blue light, and then followed by X-ray irradiation 24 h later. Cells were harvested for the apoptosis analysis, 2 days after X-ray irradiation. The figure shows the percentage of Annexin V–positive cells. * *p* < 0.05. (**C**) HNSCC cells were irradiated with blue light, and X-rays were irradiated 24 h later. The cells were harvested for Western blotting, 2 days after X-ray irradiation. Representative immunoblots of cleaved caspase-3 are shown. GAPDH was used as a loading control.

In SAS cells, exposure to blue light for 30 min significantly increased the proportion of Annexin V–positive apoptotic cells (Figure 6A). In the combined treatment group, the percentage of apoptotic cells following 30 min of blue light plus 2 Gy irradiation was higher than that observed after 2 Gy irradiation alone; however, no significant difference was detected between the combined treatment and blue light alone. Consistently, blue light irradiation increased the expression of cleaved caspase-3, a proteolytic enzyme that executes apoptosis [17], whereas its expression in the combined treatment group was comparable to that in the blue light–only group (Figure 6C). In contrast, blue light irradiation did not induce apoptosis in Ca9-22 cells, and the apoptotic response to combined irradiation was similar to that observed following 6 Gy irradiation alone (Figure 6A,B).

These results suggest that apoptosis is not the primary mechanism responsible for the enhanced growth suppression observed following combined blue light and ionizing radiation.

### 3.6. Effects of Blue Light Irradiation Radiosensitivity of HNSCC Cells

**Figure 7:**
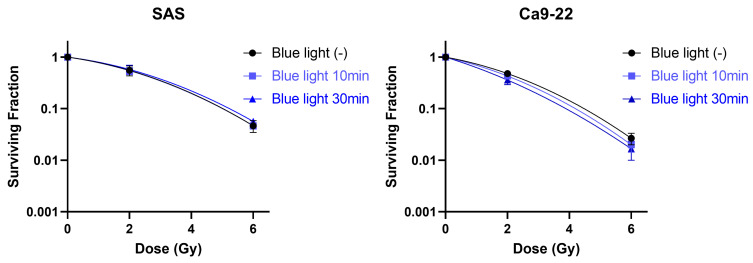
Effects of blue light irradiation on radiosensitivity of HNSCC cells. SAS and Ca9-22 cells exposed to blue light were irradiated with X-rays. The cells were harvested for the estimation of radiosensitivity by the colony formation assay, 24 h following X-ray exposure.

Next, we assessed whether blue light irradiation affects the intrinsic radiosensitivity of HNSCC cells using clonogenic survival assays. As shown in Figure 7, blue light irradiation did not significantly alter the clonogenic survival curves of either SAS or Ca9-22 cells following X-ray irradiation. These findings indicate that blue light does not enhance radiosensitivity per se and that the observed antiproliferative effects are independent of classical radiosensitization mechanisms.

### 3.7. Effects of Blue Light Irradiation and Combined Exposure to Blue Light and Radiation on HUVECs Proliferation

**Figure 8:**
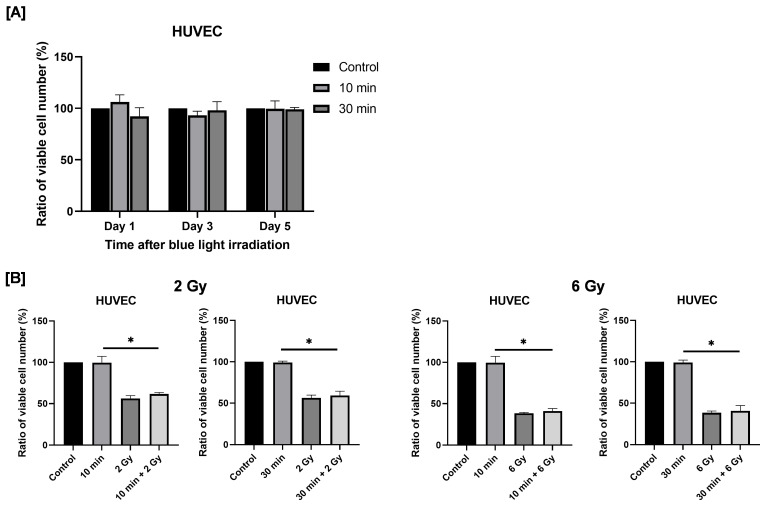
Effects of blue light irradiation and combined exposure to blue light and X-rays on HUVECs proliferation. (**A**) Each cell was irradiated with blue light for 10 or 30 min, and the number of viable cells was determined by using the trypan blue exclusion method 1 to 5 days after irradiation. The results are shown as relative values to the number of viable cells in non-irradiated cells. (**B**) HUVECs were irradiated with blue light were irradiated 24 h following blue light irradiation. The number of viable cells was counted 4 days after X-ray irradiation. The results are shown as relative values to the number of viable cells of the non-irradiated cells with blue light and X-rays. * *p* < 0.05.

To evaluate the potential effects of blue light irradiation on normal (non-cancerous) cells, HUVECs were used as a model of normal human endothelial cells, and the number of viable cells was quantified following irradiation. Here, we used HUVECs because endothelial cells represent normal cells that are universally present around tumors and are therefore highly relevant for evaluating treatment safety. Cells exposed to blue light were cultured for 1–5 days. As shown in Figure 8A, no significant differences in viable cell numbers were observed between non-irradiated and blue light–irradiated groups at any time point.

Next, we examined the effects of combined exposure to blue light and ionizing radiation on HUVEC proliferation. Irradiation with 2 or 6 Gy X-rays significantly reduced the number of viable HUVECs. Combined exposure to blue light and 2 Gy X-ray irradiation resulted in a greater reduction in viable cell numbers compared with blue light irradiation alone (Figure 8B). A similar tendency was observed following combined exposure to blue light and 6 Gy irradiation (Figure 8B).

## 4. Discussion

It has been reported that blue light irradiation exerts anticancer effects [5,18]. While some studies have investigated its use in combination with current anticancer therapies [11], the combination of blue light and ionizing radiation is yet to be fully validated. It has been shown in this study that blue light irradiation inhibited the proliferation of human HNSCC cells and that combined irradiation with blue light and X-rays effectively inhibited cell proliferation compared with each treatment alone. In particular, the combination of blue light irradiation and 2 Gy demonstrated a proliferation-inhibiting effect comparable to those of 6 Gy alone in HNSCC cell line SAS. This indicates that using combined irradiation may permit a reduction in radiation doses while still maintaining effectiveness against cancer, thus minimizing side effects in healthy tissues.

Blue light irradiation effectively suppressed the proliferation of HNSCC cells, while only minimal effects were observed in lung cancer A549 and colon cancer HCT116 cells. The reason behind the varying responses observed in different cell lines are still not fully understood; however, previous studies have indicated that blue-light-sensing photoreceptors like Opsin 3 play a role in the anticancer properties of blue light [19]. Therefore, it is important to explore the relationship between the expression levels of blue-light-sensing photoreceptors and cellular responsiveness to blue light in future research.

The anticancer effects of blue light on HCT116 cells were reported by Yoshimoto et al. [19]. However, the present study did not observe this effect. This discrepancy may be explained by differences in the energy administered to the cells. In brief, HCT116 cells were irradiated by Yoshimoto et al. with 465 nm blue light at 300 W/m^2^ for 30 min [19], whereas our irradiation condition was 470 nm blue light at 30–40 W/m^2^ for 30 min. Thus, the energy delivered to HCT116 cells in the study by Yoshimoto et al. was approximately 10 times higher than the energy in our study. Consistent with this idea, it has been shown in previous studies that the anticancer effects of blue light increase with the amount of delivered energy [11,19].

Although the anticancer effects of the combination of blue light (30 min) and 6 Gy were comparable to those of the effects of using 6 Gy alone, enhaced effects were observed with blue light (30 min) and 2 Gy. In this experiment, 6 Gy was administered in a single fraction; however, conventional radiation therapy is usually administered in daily fractions of 2 Gy. Interestingly, studies have indicated that blue light delivered in fractions more effectively induces apoptosis than when administered in a single dose [19,20]. Therefore, it is worth investigating the anticancer effects of fractionated irradiation protocols combining blue light and ionizing radiation.

Some reports indicate that blue light has anticancer properties by inducing apoptosis [21,22,23,24]. For example, Zhuang et al. reported that blue light irradiation induces apoptosis in human promyelocytic leukemia cell lines, accompanied by increased expression of cleaved caspase-3 and caspase-9 [25]. Consistent with these findings, we also found that blue light irradiation increased apoptosis and elevated the expression of activated caspase-3 in SAS cells. Therefore, induction of apoptosis is regarded as one of the mechanisms that contribute to the anticancer effects of blue light irradiation on SAS cells. However, the use of combined irradiation with blue light and radiation failed to further increase the apoptotic cells of SAS cells in comparison to the use blue light alone. This indicates that the improvement of the anticancer effects by the combination may not be related to apoptosis induction.

Of note, blue light irradiation reduced the expression levels of phosphorylated ERK1/2, which is involved in cell proliferation, as well as the intracellular ROS levels. From what we understand, this is the first report showing that blue light irradiation reduces both expression levels of phosphorylated ERK1/2 and ROS. Since ROS are known to activate ERK1/2 [26], it is likely that blue light has an influence on ERK1/2 activity through the regulation of intracellular ROS. Importantly, suppression of phosphorylated ERK1/2 by blue light irradiation was also observed in SAS cells exposed to 2 Gy of ionizing radiation. Since the use of 2 Gy alone did not reduce the expression of phosphorylated ERK1/2, it is possible that the anticancer mechanisms of ionizing radiation differ from those of blue light irradiation. This difference in mechanism might account for the enhanced anticancer effects observed when they are used in combination.

Although we found that blue light decreased intracellular ROS levels, some reports have demonstrated that blue light irradiation increases intracellular ROS levels [27,28]. For example, Yang et al. reported that blue light increases intracellular ROS levels for human osteosarcoma cell line MG63 [6]. However, blue light has been demonstrated to increase antioxidant enzymes in plant cells, but not mammalian cells, and these enzymes include superoxide dismutase, catalase, and glutathione peroxidase, which suppresses ROS levels [29]. In mouse photoreceptor cells exposed to blue light, an increase in superoxide dismutase 1 expression was observed following an initial increase in ROS levels [30]. These findings suggest that ROS levels may transiently increase immediately after blue light exposure, which triggers activation of the antioxidant system and a subsequent reduction in ROS levels. To confirm this potential, it will be essential to examine ROS levels and the expression of antioxidant enzyme both during and after blue light irradiation.

Unlike SAS cells, Ca9-22 cells did not exhibit ERK1/2 inhibition or apoptosis induction in response to blue light irradiation. As a result, the anticancer mechanism of blue light seems to differ based on the cell line, and in Ca9-22 cells, proliferation may be suppressed through pathways that do not involve ERK1/2 inhibition and apoptosis. In line with this, the effects of blue light on human osteosarcoma cells have also been shown to vary among different cell lines [6]. Although the precise mechanism in Ca9-22 cells is still unknown, programmed cell death encompasses multiple forms beyond apoptosis [31,32,33]. For example, short-wavelength blue light has been reported to induce pyroptosis in lens epithelial cells via caspase-1 activation [34], and it has also been demonstrated to trigger ferroptosis, which is an iron-dependent lipid peroxidation–driven form of programmed cell death [35]. Therefore, clarifying the anticancer mechanism of blue light in Ca9-22 cells will necessitate exploring types of programmed cell death other than apoptosis.

In this study, blue light irradiation at 470 nm, which inhibited cell proliferation in HNSCC cells, did not affect the proliferation of HUVECs. Previous studies have reported that blue light can influence cell proliferation in HUVECs. For example, Kan et al. demonstrated that blue light at a wavelength of 453 nm affected HUVEC proliferation [36]. In their study, irradiation at 50–250 W/m^2^ slightly promoted or had no effect on cell proliferation, whereas irradiation at 400 W/m^2^ resulted in a significant decrease in cell proliferation [36]. In contrast, the irradiation intensity used in the present study was 30–40 W/m^2^, which is approximately one-tenth of the intensity associated with reduced cell survival in their report. Therefore, the absence of proliferation inhibition in HUVECs observed in this study is likely due to the relatively low intensity of blue light employed. Furthermore, as the biological effects of blue light have been shown to vary depending on wavelength [35], differences in the wavelength of blue light used may also have contributed to these observations. Further studies are required to clarify these issues.

This study has several limitations. First, the mechanistic analysis focused mainly on ERK1/2 phosphorylation, and upstream regulators (Ras/Raf) and downstream effectors (such as c-Myc and Cyclin D1) were not examined. Functional perturbation experiments using ERK inhibitors or activators were also not performed. These analyses will be essential in future work. Second, no direct assessments of DNA damage, DNA repair kinetics, or cell-cycle checkpoint activation were conducted. Therefore, the present data do not allow conclusions regarding enhanced radiosensitivity at the level of DNA damage or the feasibility of radiation dose reduction. The antiproliferative effects observed in this study should be interpreted strictly within the context of in vitro cellular responses. Third, the blue-light device used in this study has a fixed output intensity, preventing dose–response or time-course optimization of the treatment conditions. Finally, although we confirmed the reproducibility of the combined treatment effect in two HNSCC cell lines and the lack of toxicity in normal endothelial cells, the findings are limited to in vitro experiments. Future studies using adjustable irradiation devices, detailed molecular analyses, and in vivo models will be necessary to further validate the therapeutic potential of combining blue light with radiation.

## 5. Conclusions

In this study, we demonstrated that blue light suppresses the proliferation of HNSCC cells, and this effect was further enhanced when combined with ionizing radiation. Detailed molecular analysis revealed that suppression of the ROS–ERK1/2 pathway was observed specifically in SAS cells, suggesting that this mechanism may not be universal across all HNSCC cell types. Therefore, additional studies using other HNSCC cell lines are required to determine whether ERK1/2 suppression represents a common response to blue light. Moreover, further mechanistic investigations and evaluations of the effects of combined irradiation on normal cells and tissues will be essential to establish the therapeutic potential of combining blue light with radiation.

## Data Availability

The original contributions presented in this study are included in the article. Further inquiries can be directed to the corresponding author(s).

## Abbreviations

The following abbreviations are used in this manuscript:

HNSCC	Head and neck squamous cell carcinoma
PI	Propidium iodide
FBS	Fetal bovine serum
DMSO	Dimethyl sulfoxide
ROS	Reactive oxygen species
ERK1/2	Extracellular signal-regulated kinase
